# Consistency Rates of Clinical Diagnosis and Histopathological Reports of Oral Lesions: A Retrospective Study

**DOI:** 10.5681/joddd.2014.020

**Published:** 2014-06-11

**Authors:** Shirin Fattahi, Sepideh Vosoughhosseini, Monir Moradzadeh Khiavi, Samira Mostafazadeh, Azhdar Gheisar

**Affiliations:** ^1^Assistant Professor, Department of Oral and Maxillofacial Pathology, Faculty of Dentistry, Tabriz University of Medical Sciences, Tabriz, Iran; ^2^Professor, Department of Oral and Maxillofacial Pathology, Faculty of Dentistry, Tabriz University of Medical Sciences, Tabriz, Iran; ^3^Associate Professor, Department of Oral and Maxillofacial Pathology, Faculty of Dentistry, Tehran University of Medical Sciences, Tehran, Iran; ^4^Post-graduate Student, Department of Oral and Maxillofacial Pathology, Faculty of Dentistry, Tabriz University of Medical Sciences, Tabriz, Iran; ^5^Dentist, Faculty of Dentistry, Tabriz University of Medical Sciences, Tabriz, Iran

**Keywords:** Oral diagnosis, oral cavity, lichen planus

## Abstract

***Background and aims.*** A correct diagnosis is the most important step in the treatment of oral lesions and each oral lesion has specific clinical features that provide clinical diagnosis; however, some of these features are common among different lesions. In these situations, biopsy and histopathological examination are indicated. The aim of this study was to evaluate the relationship between clinical and histopathological diagnosis of patients referred to the Department of Oral Pathology, Tabriz Faculty of Dentistry, from 2009 to 2011.

***Materials and methods.*** In this retrospective study, individual data and clinical findings of 311 patients who had been referred to the Department of Oral Pathology during a three-year period were collected through questionnaires and compared with histopathological reports. Data were analyzed by using chi-squared and Fisher's tests.

***Results.*** In 80.7% of the cases the clinical diagnosis of the lesions was consistent with pathology reports. In 19.3% of the cases, the clinical diagnosis of the lesions was not confirmed histopathologically. The greatest consistency was observed for lichen planus (100%) and inflammatory fibrous hyperplasia (epulis fissuratum) (94.3%).

***Conclusion.*** Although great consistency rates were observed in this study between clinical diagnoses and pathology reports, there was also a significant disagreement with the literature, indicating that comprehensive clinical examination, high consistency with oral lesion features and effective cooperation between surgeons and pathologists are necessary.

## Introduction

Exact diagnosis is the most important part of treatment.^1^ Each oral lesion has specific clinical features that provide clinical diagnosis, but some of these features are common between different lesions. In these situations, biopsy and histopathological examination are indicated.^2^ Comprehensive clinical examination includes the patient's medical history, physical examination (inspection, palpation, percussion, auscultation) and using paraclinical tests if necessary to confirm or rule out some clinical diagnoses.^2,3^ Biopsy is the most common and determinant of paraclinical tests.^4^ Although the histopathological diagnosis is considered the basis for treatment of most lesions, some of the microscopic criteria are not pathogonomonic. Therefore, between cooperation between the surgeon and pathologist and correlation between comprehensive radiographic and clinical evaluations are essential to reach a definitive diagnosis.^5^

The correspondence of clinical diagnosis and histopathological reports has been evaluated in many studies. Hosseinpoor et al reported that 81.2% of clinical diagnoses were consistent with histopathological reports. The highest concordance was observed for lichen planus, inflammatory fibrous hyperplasia and leukoplakia whereas pemphigus, SCC and systemic lupus erythematous exhibited the lowest concordance.^2^ In Ghasemi's study the highest agreement rate was observed with lichen planus and mucocele.^3^ In another study Ashkavandi et al reported the same results, with the highest percentage of agreement for mucocele and reactive soft tissue lesions.^5^In the present study the consistency rate between clinical diagnoses and histopathological reports of patients referring to the Department of Oral Pathology, Tabriz Faculty of Dentistry was evaluated during a three-year period.

## Materials and Methods

In this descriptive retrospective study, 311 patient records from the archives of the Department of Oral Pathology were selected and data on age, gender, anatomic site and clinical diagnosis were collected through questionnaires. Records without exact histopathological reports or clinical diagnosis were excluded from this study. Data were analyzed by SPSS/15 software program using chi-squared and Fisher's tests. Statistical significance was defined at P ≤ 0.05.

## Results

In the review of 311 patient records, the study sample consisted of 171 (55%) females and 140 (45%) males. Female subjects ranged between 5 and 84 years of age (with a mean age of 40) and male subjects were 1–86 years old (with a mean age of 42). 70% of the lesions were peripheral and 30% were central.

Comparison of clinical and histological diagnoses showed that 251 (80.7%) of clinical diagnoses were coincident with histopathological reports but in 60 (19.3%) of the samples, the clinical diagnoses were not confirmed histopathologically.

As summarized in Table 1, the highest percentage of consistency was observed for lichen planus (100%), inflammatory fibrous hyperplasia (94.3%) and periapical cysts (83.3%).

**Table 1:** Frequency distribution (percent) of consistency rates between clinical findings and histopathological reports based on lesions

Pathologic reports	Clinical Diagnosis	
	Correct	Incorrect
Lichen Planus	25 (100%)	—
Inflammatory fibrous hyperplasia	33 (94.3%)	2 (5.7%)
Periapical cyst	20 (83.3%)	4 (16.7)
Giant cell granuloma	27 (79.4%)	7 (11.7%)
Pyogenic granuloma	18 (78.3%)	5 (21.7%)

## Discussion

Considering the importance of accurate diagnosis in proper treatment, and non-pathognomonic features of all the lesions, the coincidence between clinical and histological diagnosis seems to be important.^1,2^The main objective of this study was to adjust the clinical characteristics of the lesion in order to enable pathologists to obtain appropriate samples. This concept would assist surgeons immensely in taking proper specimens and pathologists in reaching an accurate histopathological diagnosis; each failure in the clinical diagnosis might lead to pathological diagnosis failure as well.

The present study revealed that in 80.7% of cases the clinical diagnoses were consistent with histopathological reports. This consistency rate is higher than those in Ghasemi's, Hashemipoor's and Macan's studies^3,6,7^ and less than those in Hosseinpoor's and Jaafari's.^2,5^

The difference between consistency rates can be explained by differences in proficiency of the surgeon and the pathologist, accuracy of the biopsy, manner of transfer to the laboratory, fit cut of sample and attention and quality of surgeon‒pathologist cooperation.

With respect to age, the highest percentage of agreement rate was observed in the 7th decade and older than that, consistent with other similar reports.^2,3,5,6,8^ The main reasons for this result might be the loss of teeth and a decrease in the number of lesions which originate from them. Another reason might be exclusion of lesions that develop in children or young adults.

The highest percentage of lesions was found in the gingiva and the lowest in the floor of the mouth, consistent with other reports.^2,3,5,6,8^ In Deihimi's study the greatest consistency rate was observed in vestibular and labial mucosa.^8^Although, inflammatory fibrous hyperplasia is the most common lesion in these areas and can be easily diagnosed clinically, their findings are obvious.

The third common lesion in the present study was lichen planus which also had the greatest consistency rate, consistent with Hosseinpoor's study.^2^

Since the Department of Oral Medicine is one of the main specialized centers for lichen planus it was the third most common lesion in our files and it also had the greatest consistency rate (100%).

## Conclusion

According to these results, more knowledge, attention and education, exact clinical examination and more surgeon‒pathologist cooperation are necessary for the best diagnosis and treatment. Practitioners should be encouraged to carefully investigate oral lesions in order to decrease disagreement rates between clinical diagnoses and histopathological features. Any discrepancy can lead to misdiagnosis.
